# Automatic Labeling of Vertebral Levels Using a Robust Template-Based Approach

**DOI:** 10.1155/2014/719520

**Published:** 2014-07-15

**Authors:** Eugénie Ullmann, Jean François Pelletier Paquette, William E. Thong, Julien Cohen-Adad

**Affiliations:** ^1^Institute of Biomedical Engineering, Polytechnique Montreal, Montreal, QC, Canada H3T 1J4; ^2^Functional Neuroimaging Unit, CRIUGM, Université de Montreal, Montreal, QC, Canada H3W 1W5

## Abstract

*Context*. MRI of the spinal cord provides a variety of biomarkers sensitive to white matter integrity and neuronal function. Current processing methods are based on manual labeling of vertebral levels, which is time consuming and prone to user bias. Although several methods for automatic labeling have been published; they are not robust towards image contrast or towards susceptibility-related artifacts. *Methods*. Intervertebral disks are detected from the 3D analysis of the intensity profile along the spine. The robustness of the disk detection is improved by using a template of vertebral distance, which was generated from a training dataset. The developed method has been validated using T_1_- and T_2_-weighted contrasts in ten healthy subjects and one patient with spinal cord injury. *Results*. Accuracy of vertebral labeling was 100%. Mean absolute error was 2.1 ± 1.7 mm for T_2_-weighted images and 2.3 ± 1.6 mm for T_1_-weighted images. The vertebrae of the spinal cord injured patient were correctly labeled, despite the presence of artifacts caused by metallic implants. *Discussion*. We proposed a template-based method for robust labeling of vertebral levels along the whole spinal cord for T_1_- and T_2_-weighted contrasts. The method is freely available as part of the spinal cord toolbox.

## 1. Introduction

Magnetic resonance imaging (MRI) of the spinal cord has tremendous potential for improving diagnosis/prognosis in neurodegenerative diseases and trauma as well as for developing new drugs. In particular, multiparametric MRI, which combines several semiquantitative techniques (e.g., diffusion-weighted imaging, magnetization transfer, and functional MRI), provides a variety of biomarkers sensitive to white matter integrity and neuronal function [1, 2]. However, spinal cord MRI in research and clinics is underutilized, a direct consequence of the difficulties related to the numerous artifacts and low signal sensitivity in the spine region. Even though recent developments in phased-array coil, acquisition protocols, and processing techniques helped overcoming these challenges, more efforts could be put toward making these developments available to the broad community of researchers and clinicians. A major aspect that slows down the application of spinal cord MRI in research and clinics is the lack of a standard processing pipeline, which prevents researchers from validating new developments and applying them to clinical studies.

Currently, the standard method to quantify metrics in the spinal cord is to manually draw regions of interest (ROI) in specific subquadrants of the cord and then average each metric for specific vertebral levels [1, 3–8]. One disadvantage of this method is that it is relatively time consuming and sensitive to operator bias.

In order to automate this procedure, the community requires methods that (i) automatically segment the spinal cord from the CSF and (ii) automatically label vertebral levels. While several segmentation methods have already been developed [9, 10], only very few studies have focused on automatic labeling of the vertebrae based on MRI data [11, 12].

To identify intervertebral disks, the method by Peng et al. builds a polynomial curve through the center of the intervertebral disks by convolving the spine MR image with a disk model [12]. Then, the sagittal slice with the lowest disks heights variance is selected. The disks are identified by the maxima of the profile and adjusted by detection of the boundaries of the disk. Corso et al.'s method is based on a two-level probabilistic model taking into account the appearance and relative location of the disks in a sequence of images [11]. The two previous methods extract information from a 2D analysis, which might result in a loss of information in case of spine curvature in the right-left direction (e.g., scoliosis) or lack of robustness in case of artifacts. Moreover only T_1_-weighted MR images are usually supported by these algorithms. Indeed, T_2_-weighted images can sometimes be acquired as part of an imaging protocol. These spin echo data with relatively long echo time typically exhibit low contrast between the disks and the vertebral body, rendering the classical labeling methods less robust.

The approach by Štern et al. [13] works as follows: from the intensity and gradient profiles, the periodicity of the repeating pattern matching the disks is identified by an autocorrelation over a certain range along the rostrocaudal direction. A model of the signal profile from the vertebral body is built from the previously found periodicity. Then, the maxima of the cross-correlation between the model and the intensity profile give the disks locations. This method has the advantage of supporting any modality and contrast (computed tomography, T_1_- and T_2_-weighted MR images). However, the method was only validated for the lumbar region. The authors mentioned that parameters should be modified so that the method can perform adequately in the cervical or thoracic levels, notably due to the different sizes/distances of vertebrae.

Most of the existing methods are highly sensitive to image quality. The method by Peng et al. first identifies the disk that gives the highest intensity profile and then finds adjacent disks based on intensity criteria. Hence, if a disk has an average intensity lower than an arbitrary threshold, the disk is missed by the algorithm [12]. Štern et al.'s initialization step is based on the autocorrelation of the spine profile to detect the periodicity of the disks. Therefore, if the disk/vertebral body contrast-to-noise ratio (CNR) is low, a wrong periodicity can be captured and subsequently used for detecting other disks [13]. Therefore, all of the above methods require images with sufficient CNR and intensity homogeneity throughout the whole volume considered. Indeed, all disks should have similar intensity profile in order to be automatically detected using either cross-correlation methods [13] or arbitrary threshold [12]. This condition is however not always true due to intensity biases caused by inhomogeneous excitation and/or different coil sensitivity profile. Although methods exist to correct for intensity inhomogeneities [14], these have been validated for the brain and do not produce robust results in the cord.

Most importantly, all these methods aim at being applied to pathological cases. In presence of altered/missing disks due to trauma or susceptibility artifacts caused by metallic implants, the current methods will fail by missing one or several disks.

In light of the past studies, what is lacking is a tool that (i) can handle multiple MR contrasts (T_1_- and T_2_-weighted), (ii) performs equally well in the cervical, thoracic, and lumbar region, (iii) is robust towards altered/missing disks due to pathology or artifacts, and (iv) is made freely available to the community.

Our method is based on a 3D analysis that takes into account every spine curvature (i.e., in mediolateral and anteroposterior directions). The algorithm supports T_1_-weighted and T_2_-weighted contrasts. In case of missing/degenerated disk or low CNR, it is able to estimate a probabilistic disk location based on a template. The template was calculated from a collection of vertebral distances along the cervical, thoracic, and lumbar spine in adult humans. This template-based approach not only provides increased robustness towards low CNR and altered/missing disks but also increases the quality of disk detection in case of strong signal dropout caused by metallic implants. The software is freely available at http://sourceforge.net/projects/spinalcordtoolbox/.

## 2. Materials and Methods

In this section, we first detail the algorithm, and then we validate it in ten healthy subjects and in one patient with spinal cord injury and metallic implant.

The algorithm takes as main input a spine MR image (any vertebral range can be included). Vertebrae labeling is based on the analysis of the intensity profile along the spine. Depending on the contrast (T_1_- or T_2_-weighted), generic profile shapes are used to identify disks from their intensity profile. An original feature of this algorithm is the use of a template of human vertebral distances to increase the robustness of disk detection. Steps of the method are as follows:

1. reconstructing the spinal cord centerline,
2. building the 3D intensity profile along the spine,
3. finding the intervertebral disk separating C2 and C3,
4. detecting all other disks in the image.

### 2.1. Reconstructing the Spinal Cord Centerline

**Figure 1:**
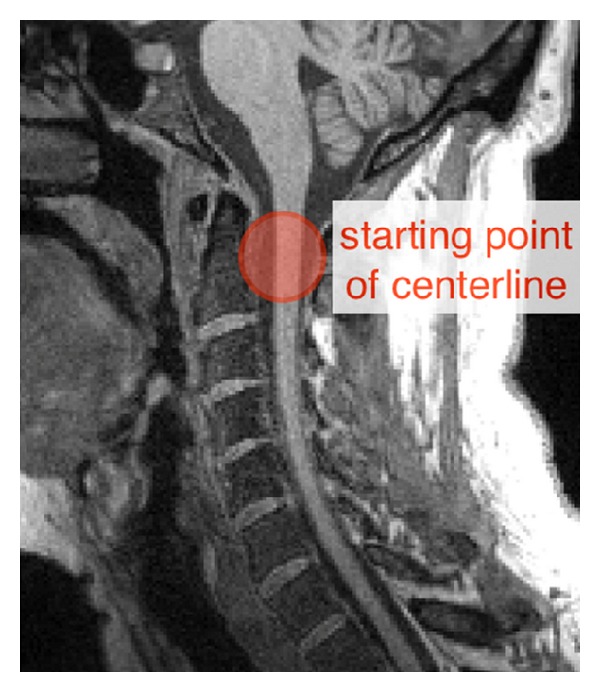
Initialization of the spinal cord centerline. In order to detect C2/C3 automatically, the centerline should begin approximately within the red circle.

The spinal cord centerline is reconstructed using the fully automatic method described in [15]. This method is based on the iterative propagation of a deformable model. The automatic vertebrae labeling requires the centerline to include the C2 vertebra area as shown in Figure 1. However, the automatic peak detection is still possible if the user indicates the level of the first visible vertebrae on the image (i.e., the most rostral one on the field of view (FOV)).

### 2.2. Building the 3D Intensity Profile along the Spine from the Spinal Cord Centerline

**Figure 2:**
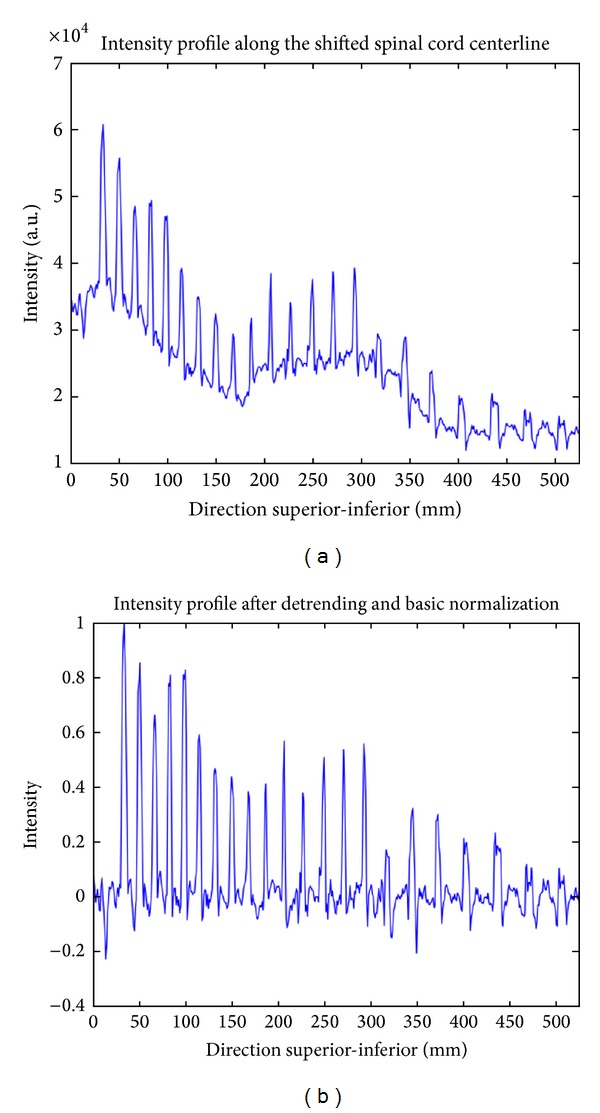
Intensity profile of a T_1_-weighted MR image before (a) and after detrending and normalization (b).

Firstly, the spinal cord centerline is shifted by 15 mm towards the anterior direction. 15 mm was chosen empirically, based on 15 subjects from preliminary data. The intensity profile along this shifted centerline is then sampled. Given that the distance between the spinal cord and the spine is variable across subjects, the intensity profile is averaged over 10 mm in the left-right direction and over 10 mm orthogonally to the centerline in the anterior-posterior direction. The baseline of the signal is removed using robust sinusoidal curve fitting. The resulting signal is then normalized by its maximum for T_2_-weighted MR image and by its minimum absolute value for T_1_-weighted MR image (see Figure 2).

### 2.3. Finding the Intervertebral Disk Separating C2 and C3

The intensity profile of a typical T_1_-weighted MR image exhibits peaks that correspond to intervertebral disks (see Figure 2). In T_2_-weighted MR images, these peaks are reversed. Peaks can be approximated by the following sinc function: pattern = sinc(space/width)^2∗*n*^, where space is a vector of 11 points centered at 0 and width is the full width at half maximum (FWHM) of the sinc function. The sinc function is powered at 2∗*n* to eliminate lobes and negative values. Based on preliminary results, we set the width default parameter at 15 mm (approximately the disk width) and *n* at 10. Note that these default values are not critically sensitive towards the robustness of the peak detection method; that is, a range of [10–20] mm for width and [8–12] for *n* will work equally well.

**Figure 3:**
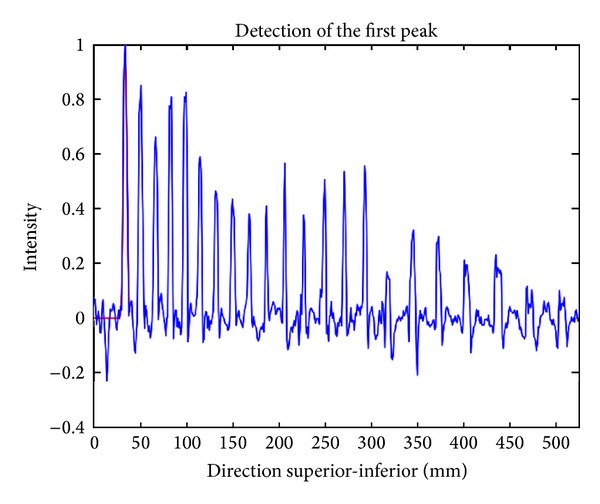
Illustration of the detection of the first disk in the image, which corresponds to the most rostral peak.

Once the sinc function pattern has been calculated, it is cross-correlated with the intensity profile (i.e., using a moving window). The first local maximum of the correlation profile (i.e., the most rostral one) corresponds to the location of the first peak (C2-C3 disk).  Figure 3 illustrates the detection of the first disk. Note that this step is only performed if the FOV includes the C2 level (see Figure 1). If not, the user has to indicate the vertebral level of the first visible vertebrae on the image.

### 2.4. Detecting All Other Disks in the Image

#### 2.4.1. Preliminary Work: Building a Template of Intervertebral Disk Distances

**Figure 4:**
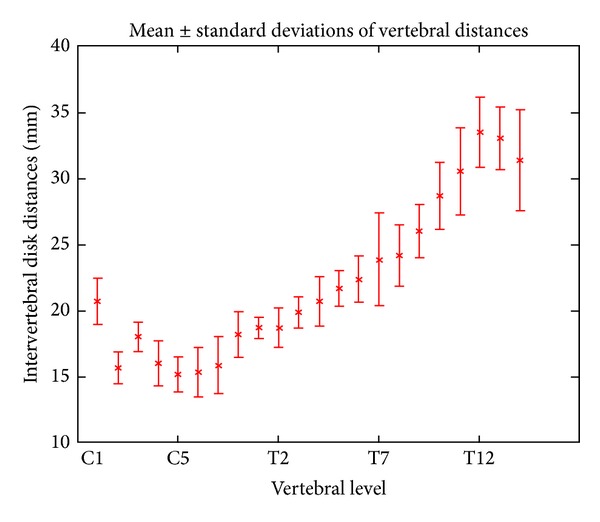
Template of generic vertebral distances. Mean distances and standard deviation of intervertebral disks in six adult subjects.

One of the main features of our method is the use of a template to increase the robustness of disk detection. A template of generic disk locations was built from six individual subjects (mean age = 29 ± 5.9 years). Disk positions were specified by hand, and then each distance between adjacent disks was measured for each subject. This distance was then averaged across subjects to give the so-called generic_distance (see Figure 4). The overall pattern of distance between disks is remarkably similar across subjects. As expected, the distance between two adjacent disks increases in the caudal direction. The larger standard deviation towards the caudal direction reflects higher variability of vertebral body sizes in the thoracolumbar spine. Each mean and standard deviation is then used as probabilistic constraint to detect disks, as described in Figure 4.

#### 2.4.2. Peak Normalization

**Figure 5:**
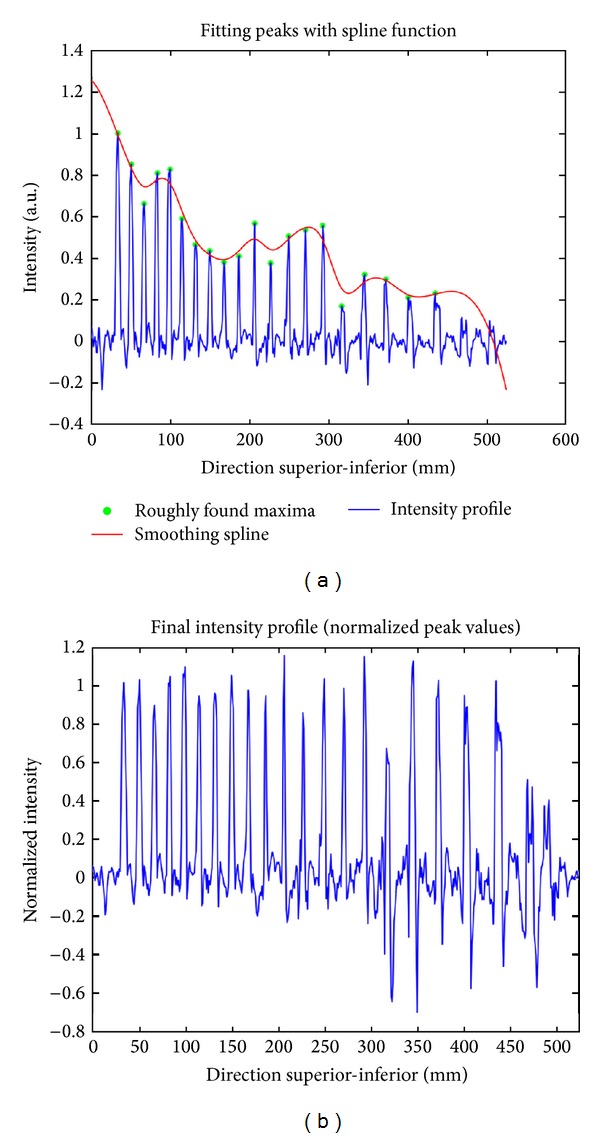
Normalization of peak heights is achieved by fitting a cubic spline function to the roughly estimated peaks (a). Then, peaks are normalized in order to achieve robust detection (b).

In order to reduce the sensitivity to intensity inhomogeneities across disk profiles, all peaks amplitudes are normalized. A cubic spline function that interpolates each local maxima is estimated (see Figure 5). Then, each intensity profile value is divided by the corresponding smoothing spline value. This normalization enables the part correction for the signal inhomogeneities and helps improve the disk's CNR which facilitates the detection. This is especially useful in the lumbar section of the spine where the peak intensities are generally lower.

#### 2.4.3. Detection of Disks

The algorithm detects disks one after another by analyzing the spine intensity profile towards the caudal direction. The starting point is the C2-C3 disk (or the disk indicated by the user). For each disk to be detected, its probabilistic location is first estimated using the template of intervertebral disk distances (see details below). Then, the probabilistic location is fine-adjusted using local cross-correlation between the intensity profile and the previously defined function pattern. Finally, all disks locations are found and then projected back to the spinal cord centerline.

**Figure 6:**
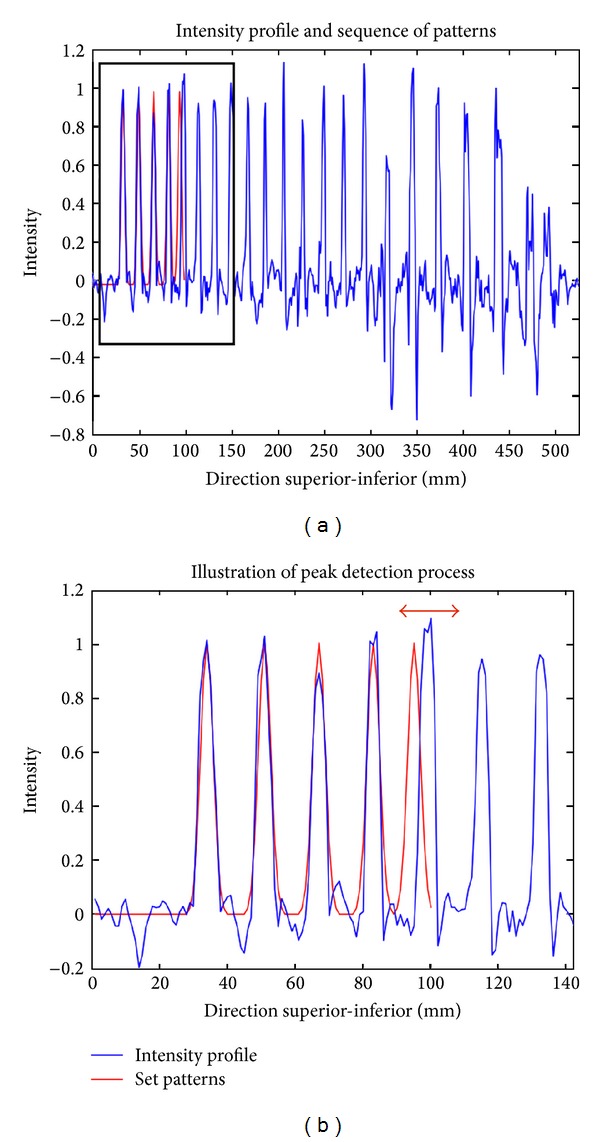
Example of peak detection (a). The blue line shows the intensity profile along the spine (after preprocessing). The red line shows the set of patterns. These patterns are based on the template and are adjusted at each new peak detection. A zoomed panel illustrates the process of peak detection (b). The last red peak on the right is being adjusted. The red horizontal arrow shows the range for which the correlation between the pattern and the profile is being computed.

The estimation of the probabilistic location of the disk uses the template of generic_distance. After each new disk detection, the vertebral level is known and the distance between the current disk and the previous disk can be iteratively estimated. The probabilistic location is calculated as follows:

(1) prob_location(i+1)=first_peak_loc⁡+∑k=1iadjusted_location(k)+generic_distance(i)∗ratio(i),

where prob_location is the probabilistic location of the (*i* + 1) disk, first_peak_location is the location of the disk separating C2 and C3 found at step 3, adjusted_location is the series of previous locations found by the algorithm, generic_distance (*i*) is the distance between the disk *i* and the disk (*i* + 1) obtained from the template, and ratio (*i*) adjusts the generic distances to everybody's own morphology: it iteratively scales the template. The variable ratio is updated for each new peak detection and is calculated by dividing the generic_distance (template-specific) by the true distance (subject-specific) between disk “1” and disk “*i* + 1.” A cross-correlation is then computed between the pattern and the intensity profile over a given range that allows describing human vertebral distance variability. The range of peak searching is within 20% of generic_distance (*i*)∗ ratio (*i*). The value of 20% has been set according to the value of standard deviation of intervertebral distances across subjects from the template (see Figure 4). The highest correlation over this range, max_corr (*i*), corresponds to the adjusted location of the peak.  Figure 6 illustrates the detection process.

**Figure 7:**
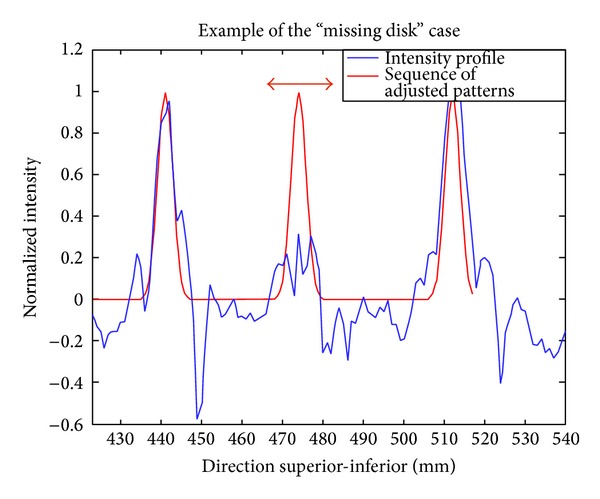
Illustration of the “missing disk” case. This figure shows the intensity profile of a T_1_-weighted image along the spine (blue) and the estimated peaks (red). The second peak has been set to prob_location because the cross-correlation between the pattern and the intensity profile was too low. This can happen in case of artifact (motion, susceptibility caused by metallic implant), low SNR, or pathology such as degenerated disk.

If the CNR between the disk and the vertebral tissue is particularly low, the highest cross-correlation value max_corr(*i*) may not be relevant and will result in wrong positioning of the disk. In such case, the algorithm sets the peak location to prob_location(*i*), which corresponds to the template-based location of the disk adjusted by the subject's own morphology (see Figure 7). The decision not to rely on the cross-correlation is made if max_corr(*i*) is inferior to 40% of the median of max_corr ([1 : *i* − 1]). The value 40% was chosen based on preliminary results and was found to work on all other tested subjects (see Section 3). 

#### 2.4.4. Projecting the Disks Position Back to the Centerline and/or the Surface

The final outputs of the algorithm are the centerline and spinal cord surface labeled with vertebral position. This labeling is found by orthogonally projecting the disks position onto the spinal cord centerline. The labeling is a numerical index corresponding to the level of the vertebrae; for example, “1” = C1, “2” = C2,…, “8” = T1, and so forth.

## 3. Results

Ten healthy subjects were recruited (mean age 30 ± 9.7 years). These subjects were different from the ones used to build the template. In addition, one patient with chronic traumatic spinal cord injury participated in this study. This patient had a metallic implant (titanium) fixed with screws at the levels C4, C5, and C6. These screws usually produce strong artifacts in gradient echo T_1_-weighted and spin echo T_2_-weighted images, typically used in clinical protocols. Experiments were performed on a 3T system (TIM Trio, Siemens Medical Solutions, Erlangen) at the* Unité de Neuroimagerie Fonctionnelle* of the* Centre de Recherche de l'Institut Universitaire de Gériatrie de Montréal*. Informed consent was obtained from all participants and the project was approved by the Comité mixte d'éthique de la recherche du Regroupement Neuroimagerie/Québec. The performance of the proposed method was evaluated on 8 T_1_-weighted (MPRAGE sequence) and 8 T_2_-weighted (SPACE sequence) MR images.  Table 1 lists some images acquisition parameters.

### 3.1. Computation Time

The software was tested on a Mac computer (dual-core i5, 2.5 GHz, 8 GB RAM) and runs in less than a minute.

### 3.2. Accuracy

Correct vertebral labeling was assessed by comparing the vertebral levels given by the algorithm and the vertebral labeling identified by an experienced user. For each modality (T_1_-weighted and T_2_-weighted) and each subject, accuracy was 0% if at least one vertebra was mislabeled and 100% if all vertebrae were correctly labeled. Then, accuracy was averaged across healthy subjects (*N* = 11). Results show an accuracy of 100% for T_1_-weighted images and 100% for T_2_-weighted images.

### 3.3. Precision

**Figure 8:**
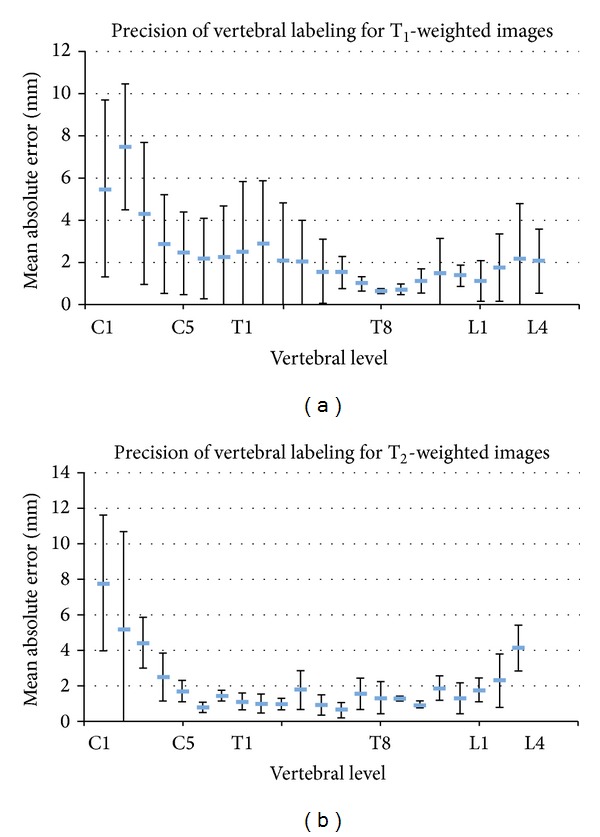
Precision of vertebral labeling for T_1_- and T_2_-weighted images at each vertebral level. The plots show the absolute errors averaged across subjects and raters. Error bars represent the standard deviation.

**Figure 9:**
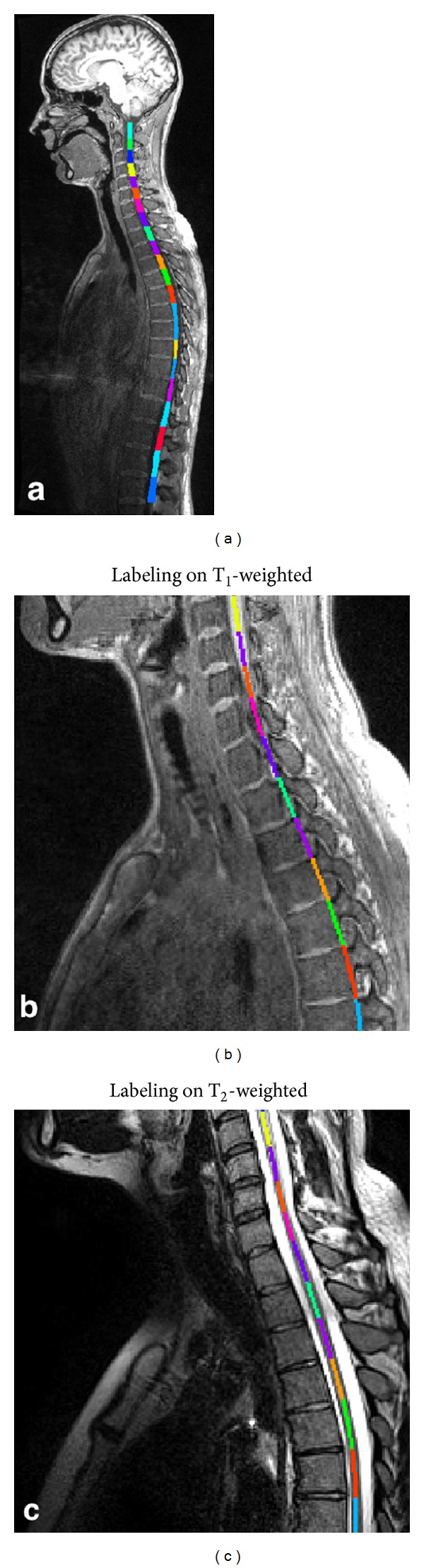
The left panel (a) shows T_1_-weighted MRI with an overlay of the labeled segmented spinal cord surface between C2 and L2. A zoomed panel shows the labeled centerline between C4 and T2 vertebrae for the T_1_-weighted (b) and the T_2_-weighted images (c).

Errors on the intervertebral boundaries were assessed. For each image, disk locations were manually identified by two experienced users. The absolute error between the automatic and the manual labeling was calculated for each user, each vertebral level, each modality, and each subject.  Figure 8 shows the mean and standard deviation across users and subjects. The mean and standard deviation of the absolute error across users, vertebral levels, and subjects was 2.3 ± 1.6 mm for T_1_-weighted images and 2.1 ± 1.7 mm for T_2_-weighted images.  Figure 9 shows the labeled segmented surface and centerline.

### 3.4. Robustness towards Metallic Implant

**Figure 10:**
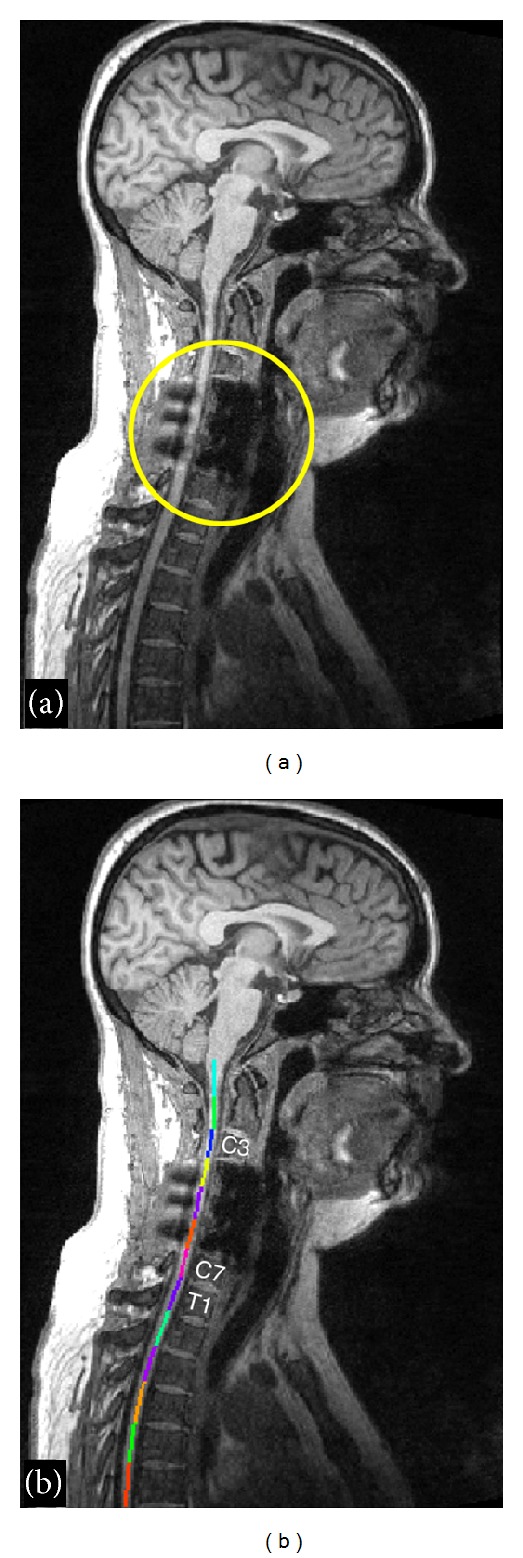
Automatic vertebral labeling in a patient with metallic implant. Large hypointensity due to dropout artifact is visible at levels C4–C6 anteriorly and is caused by the metallic screws (a). Thanks to the template-based approach, labeling was nevertheless successful (b).

To illustrate the robustness of the algorithm towards image artifacts or low CNR, the method has been tested on a patient with traumatic spinal cord injury. This patient had a metallic implant (titanium) fixed with screws at the levels C4, C5, and C6. This type of implant induces strong signal dropout in T_1_-weighted gradient echo images and T_2_-weighted spin echo images.  Figure 10(a) illustrates the dropout on a T_1_-weighted image and Figure 10(b) shows the result of the labeled centerline. Even though the artifact extends throughout three adjacent vertebral levels, the centerline appears to have been correctly reconstructed. Moreover, the labeling was 100% accurate, despite the strong signal dropout within the spine, yielding almost no contrast between the disks and the vertebral bodies at levels C4, C5, and C6.

## 4. Discussion

In this paper, an algorithm has been developed for the automatic labeling of vertebral levels on T_1_- and T_2_-weighted MR images. After building an intensity profile along the spine, disk locations are detected using a combination of local cross-correlation values and probabilistic information from a template generated from six adult subjects. Original features are (i) the possibility to use T_1_-weighted and T_2_-weighted contrasts, (ii) the possibility to include the cervical, thoracic, and/or lumbar region, and (iii) the use of a probabilistic template that increases the robustness towards altered/missing disks due to pathology or artifacts.

### 4.1. Template-Based Approach

The generic characteristics of the vertebral body have already been exploited for image segmentation in previous works. Statistical template of vertebra shape has often been used for segmentation with active shape models [16]. Rasoulian et al. created a multivertebrae anatomical shape, which takes into account vertebrae pose in order to increase vertebrae detection robustness [17]. These algorithms only support CT images [16] and dual-energy X-ray absorptiometry images [17]. Moreover, they were introduced within the field of spine research, whereas in the present paper, the goal is to automate the extraction of spinal cord metrics from multiparametric MRI. Therefore, our application does not require complex modeling of vertebra shape and hence can be simplified to achieve robust segmentation and fast computational time (less than a minute). To improve labeling in MR images, a statistical intervertebral distance model of the lumbar region showing no significant difference in distances across vertebrae was previously introduced [18]. However, the method by Koh et al. only uses the mean value of the intervertebral distance as a standard reference. Our method uses the characteristic evolution of intervertebral distances along the spine, not just as a reference parameter but as the basis for the disk detection. Moreover, the generic distances extracted from the template are weighted by a ratio that is iteratively estimated during the disk detection process. We demonstrated the robustness of labeling towards missing disk/artifact thanks to this template in a patient with spinal cord injury (Figure 10).

Given that this template was estimated from adults, it may not be adequate in pediatric population. To address this issue, our software provides an interface to build a study-specific template.

### 4.2. Precision

The precision of the method was evaluated by asking experienced users to label vertebral levels directly on the spinal cord, with each label corresponding to the level of an intervertebral disk. This procedure was done for allowing direct calculation of the absolute error between the automatically and the manually labeled vertebrae. Qualitatively, the labeling was precise from C1 to L5. The largest errors were observed at C1 and C2 levels, due to the fact that these vertebrae are merged and yield low disk contrast. Mean absolute error was 2.3 mm and 2.1 mm, for T_1_- and T_2_-weighted images, respectively. The smaller error and smaller intersubject variation on the T_2_-weighted scans is possibly related to the high sampling efficiency of the T_2_ SPACE sequence thanks to the short refocusing pulse trains, yielding less sensitivity to motion and flow artifacts. Corso et al. found an average error of 2.6 mm for the labeling in the lumbar region of T_2_-weighted MR images [11]. Štern et al. reported an average error of 2.9 ± 1.7 mm [13]. However, in this context, it should be noted that high precision is not critical, as one of the main applications of automatic labeling is to quantify multiparametric MRI metrics at given vertebral levels [1, 8]. In such application, the labeling is mostly used for pooling data from several patients into common group statistics; therefore precision is not the main concern. Moreover, the mismatch between vertebral and spinal levels [19] adds to the lack of precision of vertebral-based labeling methods when it comes to computing microstructural metrics or performing functional MRI experiments.

## 5. Conclusion 

This paper presented a method for automatic labeling of vertebral levels from MR images. An original feature is the use of a probabilistic template that increases the robustness towards altered/missing disks due to pathology or artifacts, as demonstrated in a spinal cord injured patient with metallic implant. The method works as standalone software and can be plugged into pipelines for extracting metrics from multiparametric MRI protocols. The software is available as part of the spinal cord toolbox: http://sourceforge.net/projects/spinalcordtoolbox/.

## Figures and Tables

**Table 1:** Acquisition parameters for the tested spine MRIs. Voxel size was 1 × 1 × 1 mm^3^.

Contrast	Image size (voxel)	Visible vertebrae (range)	Subject
T_1_	264∗684∗174(#)	C1–L3	Subject 1
T_1_	272∗732∗172(#)	C1–L2	Subject 2
T_1_	265∗633∗174(#)	C1–L3	Subject 3
T_1_	268∗757∗166(#)	C1–L3	Subject 4
T_1_	264∗690∗174(#)	C1–L3	Subject 5
T_1_	265∗679∗173(#)	C1–L3	Subject 6
T_1_	264∗384∗175	C1–T7	Subject 7
T_1_	264∗384∗175	C1–T6	Subject 8
T_2_	384∗384∗52	C1–T5	Subject 8
T_2_	384∗384∗52	C1–T3	Subject 9
T_2_	384∗692∗51(#)	C1–L3	Subject 5
T_2_	384∗384∗144	C1–T2	Subject 3
T_2_	384∗384∗52	C1–T3	Subject 7
T_2_	386∗678∗51(#)	C1–L1	Subject 6
T_2_	288∗512∗52(#)	C1–T6	Subject 10
T_2_	384∗384∗52	C1–T7	Subject 11

(#) Two fields of view were acquired sequentially and then images were stitched together using offline reconstruction tools from the console.
